# Mixed-species association and a record of a hybrid offspring between *Trachypithecus pileatus* and *Trachypithecus phayrei* in Bangladesh

**DOI:** 10.1007/s10329-022-01035-8

**Published:** 2022-11-16

**Authors:** Hassan Al-Razi, Auritro Sattar, Marjan Maria, Chonchol Guala, K. A. I. Nekaris

**Affiliations:** 1 Bangladesh Slow Loris Research and Conservation Project, Dhaka, Bangladesh; 2 Plumploris Eingetragener Verein, Mergelteichstraße 80, Dortmund, Germany; 3 Jahangirnagar University School and College, Savar, Dhaka, Bangladesh; 4 Department of Zoology, Faculty of Life and Earth Sciences, Jagannath University, Dhaka, Bangladesh; 5 Creative Conservation Alliance, Turtle Conservation Center, Bhawal National Park, Gazipur, Bangladesh; 6 Nocturnal Primate Research Group, School of Social Sciences, Oxford Brookes University, Oxford, UK

**Keywords:** Conservation, Hybridization, Langur, Primates

## Abstract

The term mixed-species association has a broad range of definitions, from temporary foraging association to permanent group living. A mixed-species association mostly involves species from closely related taxa and is found in birds, mammals and fish. It ranges from passive association with little interaction to coordinated behavioural interactions between the group members of a mixed-species group. Mixed-species association can result in the production of hybrid offspring in the wild. In this study, we present, to the best of our knowledge, the first observational evidence of mixed-species association between the two threatened primate species Phayre’s langur (*Trachypithecus pileatus*) and capped langur (*Trachypithecus phayrei*), in fragmented forest patches of northeast Bangladesh. We also report a presumed hybrid offspring between these species. We conducted a short-term study from December 2021 to April 2022 in three forest patches based on information from eco-tourism guides. We confirmed the presence of three mixed-species troops; in two of the groups an adult male *T. phayrei* had permanently immigrated into a group of *T. pileatus*, and in the other one an adult male *T. pileatus* had permanently immigrated into a group of *T. phayrei*. A long-term detailed study is needed to elucidate the reasons for these mixed-species associations, their behavioural patterns, the fate of the presumed hybrid offspring, and to understand the genetic relatedness between the individuals.

## Introduction

Although most animal groups contain members of a single species (Alexander 1974; Morse 1977; Krause and Ruxton 2002; Majolo and Huang 2018), sometimes individuals of other species join a group. This phenomenon is termed a polyspecific, heterospecific or mixed-species group. Found in birds, mammals and fish, these mixed-species groups often comprise species from closely related taxa, i.e. the same genus or family, with some exceptional cases where groups involve members from different orders or even classes (Powell 1985; Heymann and Buchanan-Smith 2000; Stensland et al. 2003; Rehg 2006; Kelm et al. 2021; Lhota et al. 2022). The interaction between the group members of mixed-species groups varies from passive association with little interaction to coordinated behaviours (Kelm et al. 2021). In primates, coordinated behavioural interactions tend to characterize mixed-species troops (Peres 1992; Heymann and Buchanan-Smith 2000l). Indeed, stable interspecific associations have been regularly recorded in guenons (*Cercopithecus* spp.) in Africa (Cords 1987; Gautier-Hion 1988), tamarins (*Saguinus* spp.) in South America (Terborgh 1983; Garber 1988), and langurs (*Semnopithecus* spp., *Trachypithecus* spp.) and macaques (*Macaca* spp.) in Asia (Lu et al. 2021; Heng et al. 2010; Choudhury 2008). There is supporting evidence that primates form mixed-species groups to gain the benefits of a larger group, such as predator defence and ecological knowledge, often without the disadvantage of increased competition for food (Chapman and Chapman 2000; Wolters and Zuberbühler 2003).

Inter-species hybridization occurs across many closely related primate species (Lu et al. 2021). Distinct natural hybridization lineages have been found in over 10% of primate species (Arnold and Meyer 2006; Zinner et al. 2011). Most hybridization evidence has come from matings between two distinct species, based on observations of mixed-species groups and reports of intermediate external morphological features in hybrid zones where closely related species live in sympatry (e.g. Dunbar and Dunbar 1974; Terborgh 1990; Phillips-Conroy et al. 1991; Aguiar et al. 2008; Lu et al. 2021). Molecular studies have also shown ancient hybridization events across different primate species (Roberts et al. 2010; Roos et al. 2011).

Phayre’s langur (*Trachypithecus pileatus*) and capped langur (*Trachypithecus phayrei*) are two of the ten primate species found in Bangladesh. Both species are globally threatened, with *T. phayrei* listed as Endangered and *T. pileatus* as Vulnerable [International Union for Conservation of Nature (IUCN) 2015]. The range of these species overlaps in Bangladesh, northeast India and Myanmar, where they also have similar habitat requirements (Das et al. 2020; Chetry and Ahmed 2021; Al-Razi and Naher 2021). In Bangladesh, the two species co-exist in evergreen and semi-evergreen forest patches (IUCN 2015). To the best of our knowledge, there are no published reports of mixed-species association or hybridization between these species in Bangladesh or in other parts of their range. We carried out a short-term study on these species in three forest patches of northeastern Bangladesh. Here, we report two records of permanent immigration by an adult male Phayre’s langur into a capped langur group, and one record of permanent immigration by an adult male capped langur into a Phayre’s langur group. We describe, to the best of our knowledge for the first time, the external features of a presumed hybrid juvenile male (*T. phayrei* × *T. pileatus*).

## Methods

### Study area

**Fig. 1:**
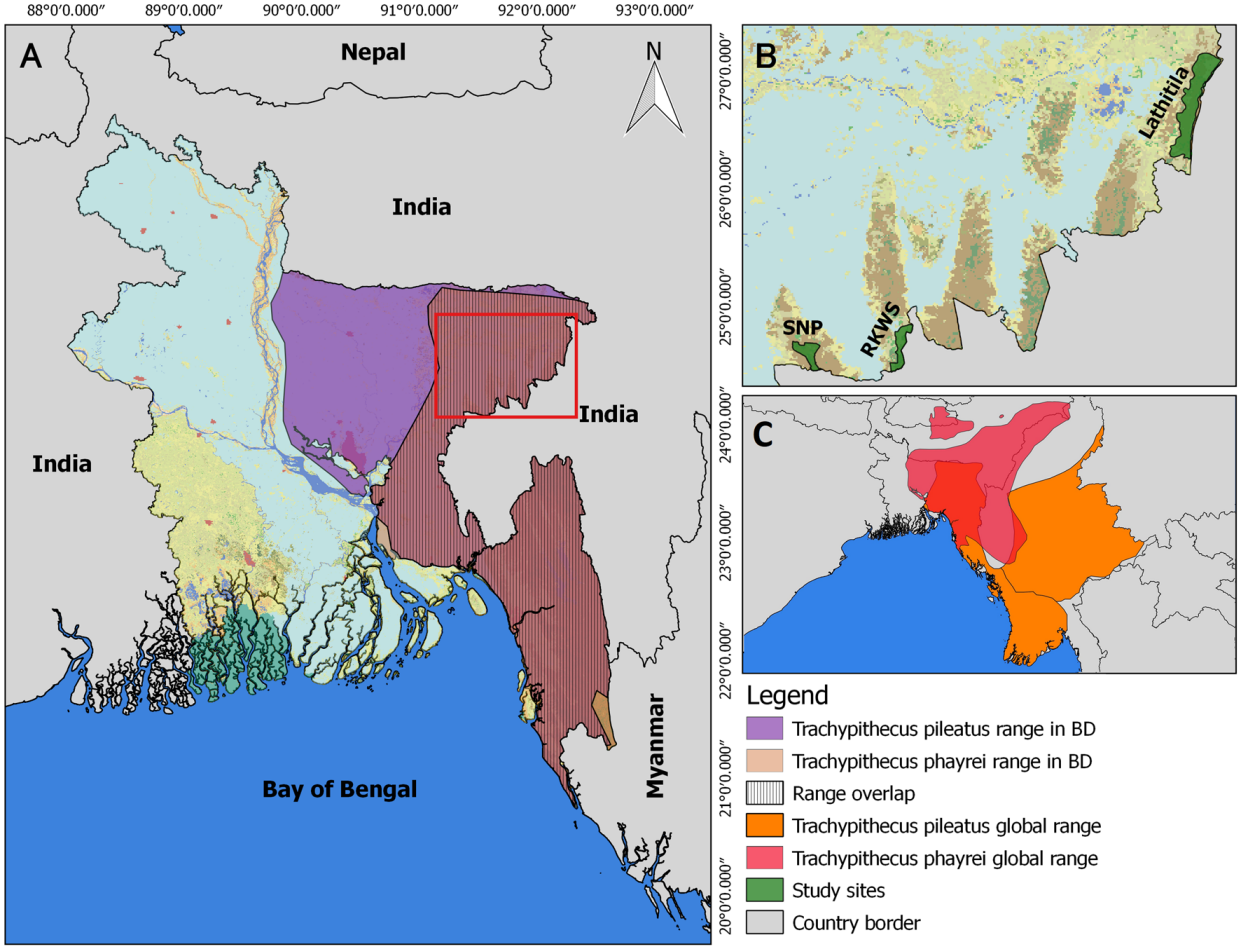
**a** Location of the study areas and range of *Trachypithecus phayrei* and *Trachypithecus pileatus* in Bangladesh (*BD*). **b** Sites of mixed-species group occurrence. **c** Global range of *T. phayrei* and *T. pileatus*

The landscape of northeast Bangladesh includes ten remaining primary forest patches, varying in size from 10 to 100 km^2^ (Bangladesh Forest Department 2012). We conducted the field study in three (Satchari, Rema-Kalenga, and Lathitila) of these ten forest patches. All the forest patches of this region are protected by law by the Bangladesh Forest Department; Satchari is a national park, Rema-Kalenga is a wildlife sanctuary and Lathitila is part of the Patharia Hill Reserve Forest (Fig. 1a, b). Satchari National Park comprises a small portion of Raghunandan Hill Reserve Forest and the wildlife sanctuary of Rema-Kalenga is situated on Tarap Hill. These forest patches are extensions of larger forest tracts in India that expand into Bhutan and Myanmar (Bangladesh Forest Department 2012). The forests vary in type from tropical evergreen to mixed and or semi-evergreen. The topography of northeastern Bangladesh is hilly, with elevations ranging from 50 to 300 m above sea level. Numerous streams crisscross the forests of this region (Rahman et al. 2021; Bangladesh Forest Department 2012). The annual temperature range is 9 to 32 °C, and nearly 80% of the annual average rainfall (3334 mm) occurs between May and October (Quazi and Tamara 2016). The forests of northeast Bangladesh harbour many globally threatened amphibians, reptiles, birds and mammals (Al-Razi et al. 2019, 2020; Zakir et al. 2020; Rahman et al. 2021). Among the ten primate species of Bangladesh, seven are found in the forest patches of the northeastern region (Al-Razi et al. 2019, 2020). Due to anthropogenic activity, the forests of northeast Bangladesh are becoming fragmented and losing their biodiversity (Reza and Hasan 2019).

### Surveys and observations

Based on camera trap surveys of canopy bridges over a highway that runs to Satchari National Park, we identified a potential mixed-species group of *T. pileatus* and *T. phayrei* (Maria et al. 2022). To confirm the mixed-species groups of *T. pileatus* and *T. phayrei*, we contacted eco-tourism guides of five forest patches of northeast Bangladesh (Satchari National Park, Lawachara National Park, Adampur Forest, Lathitila Forest and Rema-Kalenga Wildlife Sanctuary). As the eco-tourism guides often enter the forest with tourists to observe wildlife, they have a clear idea about resident animals, and a particular focus on langurs and gibbons to provide a positive experience to the tourists. We described our study goals in initial phone calls to the eco-tourism guides, who gave us the locations of potential mixed-species langur groups.

We conducted a study on a total of 27 days in the period from December 2021 to April 2022 in three forest patches (5 days in Lathitila, 7 days in Rema-Kalenga and 15 days in Satchari National Park). We surveyed human-made trails and streams. When we encountered a mixed-species langur group, we recorded the time, Global Positioning System location and the group size and composition. We classified the langurs as adult males, adult females, sub-adults, juveniles and infants based on morphological characters (Choudhury 1987; Gupta 1996, 2001). We observed groups as long as possible to record the behaviour of each individual and to understand the roles of the individuals of the different species. We could only confirm that an individual of a different species was present in groups, and then extrapolate from that information that the individual had immigrated into the group. We did not observe any migration or emigration of individuals during the study. We also observed the behaviour and external features of a presumed hybrid individual. We conducted a questionnaire survey among the people who live and/or work inside the forest to gain information about the duration of the mixed-species groups.

## Results

### Mixed-species group composition

We found three mixed-species associations in three different forest patches. In Satchari and Rema-Kalenga, one adult male Phayre’s langur appeared to have immigrated into a capped langur group. In Satchari, the group comprised eight individuals of which one was an adult male, three were adult females, two were juveniles and one was an infant capped langur and the other individual was an adult male Phayre’s langur. The juveniles were similar in size, and we could not determine their sex. The male Phayre’s langur was similar in size to the adult capped langur male of the group. This group was mostly found near human habitation at the forest edge. The mixed-species association of Rema-Kalenga comprised 12 individuals and consisted of an adult male, four adult females, four juveniles and one infant capped langur, one immigrant adult male Phayre’s langur and a presumed hybrid juvenile. According to local people and eco-tourism guides, the immigrant adult male Phayre’s langur had been living with the group of capped langurs for more than 2 years. In Lathitila, the group comprised one adult male, two adult females, two juveniles and an infant Phayre’s langur as well as an immigrant adult male capped langur (Table 1).

**Table 1:** Composition of three mixed-species langur groups in northeastern Bangladesh

Study site	Species	Adult males	Adult females	Juveniles	Infants	Hybrid	Immigrant (adult males)	Total
Satchari	*Trachypithecus pileatus*	1	3	2	1	0	1 *T. phayrei*	8
Rema-Kalenga	*T. pileatus*	1	4	4	1	1 Juvenile	1 *T. phayrei*	12
Lathitila	*Trachypithecus phayrei*	1	2	2	1	0	1 *T. pileatus*	7

We encountered these mixed-species langur groups three times in Satchari, two times in Rema-Kalenga and once in Lathitila, and were able to observe their behaviour for a total of 425 min (Satchari, 190 min; Rema-Kalenga, 165 min; Lathitila, 70 min). We did not observe any aggressive behaviour between the group members and the immigrants during our observations. In the capped langur groups, the immigrant male Phayre’s langur always kept a distance from the alpha male of the group, but interacted with other members normally. In the group in Satchari National Park, the immigrant male Phayre’s langur had affiliative interactions with a female capped langur. They groomed each other during resting, out of sight of the male capped langur. In the group in Rema-Kalenga Wildlife Sanctuary, the immigrant male Phayre’s langur interacted with all the individuals of the group except the male capped langur. The immigrant males played with all the juveniles in their groups. The presumed hybrid juvenile interacted positively with all the individuals of its group, just like the other, capped langur, juveniles. However, with respect to play, the hybrid avoided the male capped langur and preferred to play with the immigrant male. Despite dissimilarities between the external features of the presumed hybrid and the capped langur juveniles, we did not see any notable difference between them with respect to the physical distance they kept from one another.

### Description of the presumed hybrid

**Fig. 2:**
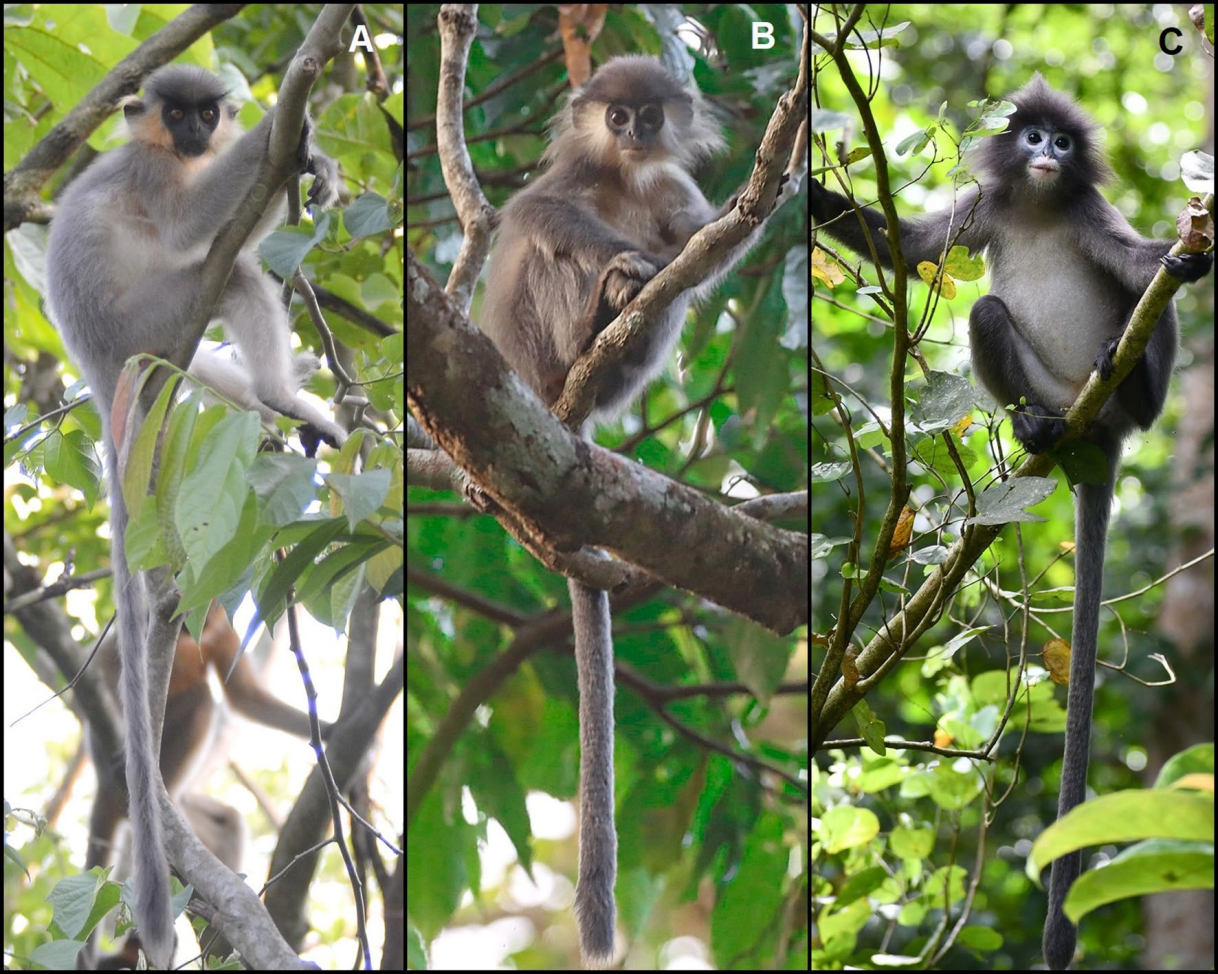
Variation in external features between **a** a juvenile capped langur, **b** the presumed hybrid juvenile langur and **c** a juvenile Phayre’s langur

**Fig. 3:**
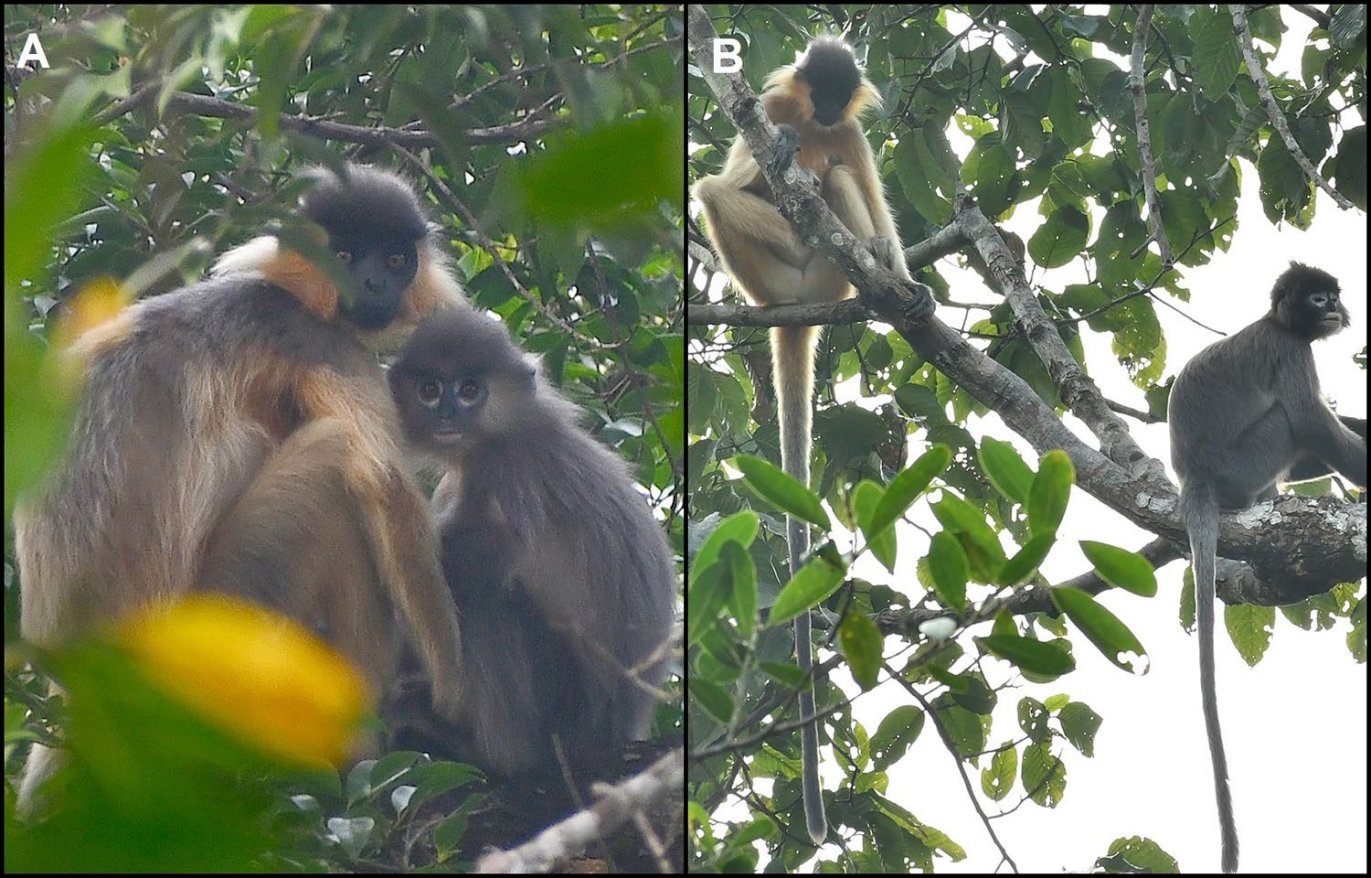
**a** The presumed hybrid sitting with his capped langur mother, and **b** the probable father (*Trachypithecus phayrei*) and mother (*Trachypithecus pileatus*) of the hybrid resting in a tree

The presumed hybrid juvenile male of Rema-Kalenga Wildlife Sanctuary had external features that resembled those of both the probable paternal (Phayre’s langur) and maternal species (capped langur). Its back was from black to ash gray in colour, which is similar to that of both its probable father and mother (Fig. 2; Fig. 3a, b). The sides of its abdomen were from pale yellow to creamy white in colour. The fur of its breast and throat region was pale yellow, while the fur of its abdomen was creamy white. The colour of its back was darker than that of a juvenile capped langur and lighter than that of a juvenile Phayre’s langur. Its head had a prominent cap, but this was darker in colour than those of the capped langur juveniles. All the fur on the tail was dark gray in colour, and the tail was thicker than those of the Phayre’s langur and capped langur juveniles. As in Phayre’s langur, there were white rings around the eyes, but these were narrow in width. Areas of the upper and lower lip region and under part of the nose were white in shade, similar to Phayre’s langur, but were narrower. This presumed hybrid had black pupils with a dark grey iris and black sclera, while its maternal species (capped langur) has black pupils, an orange iris and black sclera. In Phayre’s langur, the entire eyeball is black (Fig. 2).

## Discussion

We present here, to the best of our knowledge, the first records of mixed-species associations of primates in Bangladesh and of a probable hybrid offspring. Several cases of hybridization have been found in langurs, e.g. *Trachypithecus geei* × *Trachypithecus pileatus* (Choudhury 2008), *Semnopithecus entellus* × *Semnopithecus johnii* (Hohmann and Herzog 1985; Hohmann 1989), and *Nasalis larvatus* × *Trachypithecus cristatus* (Lhota et al. 2022). In the present study, we found an adult male immigrant in all the mixed-species groups. Lu et al. (2021) also reported an adult male immigrant, a tufted grey langur (*Semnopithecus priam thersites*), in a group of purple-faced langurs (*Semnopithecus vetulus philbricki*). An important aspect of mixed-species group formation in langurs is group size. We suggest that group size and the ages of the individuals in the new group are the main factors that influence the formation of mixed-species groups in Phayre’s langurs and capped langurs. In Satchari National Park, the immigrant adult Phayre’s langur was in a group of six capped langurs. From the group’s structure (number of individuals and age of the offspring), we assumed the group to be newly formed (Table 1). The situation was similar in Lathitila, as the male capped langur had joined what appeared to be a newly formed small group of Phayre’s langur. In Rema-Kalenga Wildlife Sanctuary, the group size was large; from our interviews with the eco-tourism guides and local people we learned that when the male Phayre’s langur had immigrated into the capped langur’s group, the group was still small. The fact that the presumed hybrid was of similar age to the four juvenile capped langurs, and thus presumably born at a similar time, indicated that the male Phayre’s langur had entered the group when it consisted of only five individuals and had subsequently mated with one of the female capped langurs.

According to Detwiler et al. (2005), natural hybridization can occur in various situations, including on the edge of a species’ range, in a fragmented habitat, or where a species is relatively rare because of interspecific competition or poor habitat quality. The western edge of the range of both capped langurs and Phayre’s langurs is in Bangladesh (Fig. 1a, c). The habitats of both species are highly fragmented and threatened by various anthropogenic activities (Hasan et al. 2018; Naher and Khan 2018; Al-Razi and Naher 2021). Thus, the fact that northeast Bangladesh is at the edge of their geographic range and comprises a highly fragmented and poor-quality habitat could potentially explain these mixed-species associations and the production of a hybrid offspring between these sympatric species. We present here, to the best of our knowledge for the from time, evidence of mixed-species association between *T. pileatus* and *T. phayrei*, and probable hybridization between langur species in this region. Long-term more detailed study is needed to elucidate the reasons for mixed-species associations, the behavioural patterns of mixed-species groups and the fate of presumed hybrid offspring. Genetic studies would also help to clarify the parentage of presumed hybrids.

## Data Availability

As it is an observation-based paper, there is no data to share.
